# A study of role expansion: a new GP role in cardiology care

**DOI:** 10.1186/1472-6963-14-205

**Published:** 2014-05-06

**Authors:** Lorraine Pollard, Stephen Rogers, Jonathan Shribman, David Sprigings, Paul Sinfield

**Affiliations:** 1 Department of Health Sciences, University of Leicester, 22 -28 Princess Road West, Leicester LE1 6TP, UK; 2 Public Health, Northamptonshire County Council, Guildhall Road, Northampton NN1 1DN, UK; 3 NHS Nene Clinical Commissioning Group, Francis Crick House, Summerhouse Road, Moulton Park, Northampton NN3 6BF, UK; 4 UK Cardiology, Northampton General Hospital NHS Trust, Cliftonville, Northampton NN1 5BD, UK

**Keywords:** GP, Primary care, Cardiology, Referrals, Extended, Role, Patients, Management (9)

## Abstract

**Background:**

The National Health Service is reconfiguring health care services in order to meet the increasing challenge of providing care for people with long-term conditions and to reduce the demand on specialised outpatient hospital services by enhancing primary care. A review of cardiology referrals to specialised care and the literature on referral management inspired the development of a new GP role in Cardiology. This new extended role was developed to enable GPs to diagnose and manage patients with mild to moderate heart failure or atrial fibrillation and to use a range of diagnostics effectively in primary care. This entailed GPs participating in a four-session short course with on-going clinical supervision. The new role was piloted in a small number of GP practices in one county in England for four months. This study explores the impact of piloting the Extended Cardiology role on the GP’s role, patients’ experience, service delivery and quality.

**Methods:**

A mixed methods approach was employed including semi-structured interviews with GPs, a patient experience survey, a quality review of case notes, and analysis on activity and referral data.

**Results:**

The participating GPs perceived the extended GP role as a professional development opportunity that had the potential to reduce healthcare utilisation and costs, through a reduction in referrals, whilst meeting the patient’s wishes for the provision of care closer to home. Patient experience of the new GP service was positive. The standard of clinical practice was judged acceptable. There was a fall in referrals during the study period.

**Conclusion:**

This new role in cardiology was broadly welcomed as a model of care by the participating GPs and by patients, because of the potential to improve the quality of care for patients in primary care and reduce costs. As this was a pilot study further development and continuing evaluation of the model is recommended.

## Background

The National Health Service (NHS) is reconfiguring health care services in order to meet the increasing challenge of providing care for people with long-term conditions and to reduce the demand on specialised outpatient hospital services by enhancing primary care [1]. Coupled with the financial constraints facing the NHS the need for effective community management of long term conditions has become increasingly important and the role of the General Practitioners (GPs) even more fundamental [2].

One proposal in the “NHS plan: a plan for investment, a plan for reform” [3] was the GP with a Special Interest (GPwSI). GPwSIs are GPs with additional experience and training in specific clinical areas that supplement their important generalist role by delivering a specialised service (e.g. respiratory care clinic) in their practice, which is beyond the traditional service provision and accepts referrals from clinicians both internal and external to their Practice [3,4].

One of the aims of GPwSIs was to reduce waiting lists for specialist services and release more specialised hospital care outpatient appointments for more complex patients. A perceived benefit of this role was its potential to improve standards in chronic disease management in primary care [4,5] and it was envisaged that these specialised primary care services would meet local health care needs [4-6]. However, in 2005, a national survey of Primary Care Trusts (PCTs) in England and Wales of the commissioning of the GPwSI role revealed that there were relatively few in post [7]. A factor for the low uptake of the GPwSI role was that it was estimated to be more costly than hospital outpatient care [8].

A county in the East Midlands of England was an early adopter of a Cardiology GPwSI role. This was one of the earliest special interest roles to be specified [9] and was subsequently underpinned by generic guidance and a formalised accreditation process [10]. The role in the first instance supported the hospital service with the GPwSI managing selected patients at a community clinic following triage by a consultant cardiologist. Later, and as part of a countywide strategy to improve the management of patients with heart failure an additional service was initiated inviting direct referral from GPs of patients with heart failure at a time when the National Institute for Health and Clinical Excellence (NICE) guidance recommended that all heart failure patients should be reviewed by a Consultant Cardiologist [11]. A significant minority of the patients referred also suffered with atrial fibrillation and with the publication of NICE guidance [12], the GPwSI managed an increasing caseload of patients where this condition was the primary diagnosis, albeit with limited impact on the ever growing level of activity within hospital clinics [13].

A review of cardiology referrals to specialised care found that General Practices hosting an individual with an interest in cardiology and up-to-date with evidence based guidelines generally referred less frequently to specialised care and had better health outcomes [14]. The combination of these findings and the key messages highlighted in the King’s Fund report on referral management [15] inspired a NHS Consultant Cardiologist and GPwSI in Cardiology in the East Midlands to develop a new in role in Cardiology. The new extended GP role was developed to enable GPs to diagnose and manage patients with mild to moderate heart failure and patients with atrial fibrillation in primary care. This was to be achieved through extending the GP’s role through participation in a brief training course and providing on-going clinical supervision. The training course provided education in elements of clinical triage, use of cardiology investigations and management of heart conditions, with associated peer review and feedback on managing cases effectively [15,16]. Previous research studies have found that brief educational interventions are beneficial to GPs’ professional development and their clinical practice, which consequently improves patient care [17].

It was envisaged that the Extended Cardiology Role GP (ECR GP) would provide an enhanced level of cardiology care within general practice and provide a practice review service for patients who might otherwise have been referred to hospital based specialised cardiology service. Whereas pre pilot the GPs would have made a referral to a hospital based Cardiologist, in the pilot ECR GPs themselves had direct access to ambulatory cardiology investigations and clinical support from a Consultant Cardiologist and GPwSI in Cardiology, so were able to manage cases without referral. In addition to “gatewaying” all non-urgent cardiology hospital referrals, the ECR GP was to become the focus of cardiology care in the practice providing clinical leadership and clinical support to their own colleagues. Advantages of the proposed extended GP role model in comparison to the GPwSI model is that the former can be rapidly implemented and disseminated at a lower cost and with the potential to make cost savings for the NHS.

The ECR GP initiative also responded to a recent opinion poll conducted by the Primary Care Trust (PCT), which indicated that patients wish to have care provided closer to home and, more specifically, to have more services provided by their local GP [18]. It is also in line with NHS policy which emphasises the importance of including the patient’s perspective in planned service development and more recently endorsed by the latest plans for the NHS contained in “Equity and excellence: Liberating the NHS” [1] which wants ‘patients at the heart of everything the NHS does”. Furthermore, current evidence suggests that clinician/patient driven quality improvement strategies may be more effective than manager/policy-maker quality improvement strategies [19].

The aim of this study was to explore the impact of the innovative ECR GP role on the GP’s role, service delivery and patients’ experience.

## Methods

GP practices in a county in the East Midlands were briefed at locality meetings inviting them to participate in the pilot and informing them of the implications. The recruited practices were selected through consultation with the PCT as being interested in cardiology management and showed enthusiasm for the aims to project. A GP from each recruited practice was nominated, from within their practice, to participate as the ECR GP in the pilot.

The participating GP Practices were offered reimbursement for the time of the GP to attend the Cardiology Education Training sessions, and for one session per week for the Cardiology in-practice service. A description of the intervention is presented in Table 1.

**Table 1:** Brief description of the intervention

**Intervention**	
The pilot ran for 21-weeks. The participating GP practices were reimbursed for the time of the GP, who attended the cardiology education training course, and for one session per week for cardiology in-practice service. There were 2 phases to the intervention, these were:	
***Educational phase***	***Clinical phase***
**Duration:** 4 sessions over 6-weeks,	**Duration:** 16 weeks in-house cardiology service.
**Tutors:** two GPwSIs in Cardiology and a NHS Consultant Cardiologist	
**Topics covered:** diagnostics in cardiology; echocardiogram, heart failure diagnosis and management; atrial fibrillation and palpitations in primary care; optimal medical management and review of clinical cases/top-up session.	**Objective:** the ECR GPs had the opportunity to implement their extended cardiology knowledge and skills. Accepted referrals on patients who presented with symptoms of heart problems at the practice.
***Clinical support***	
The ECR GPs received clinical support from a Consultant Cardiologist and the GPwSI in Cardiology. This was by telephone and by email using an encrypted email system. Contact details were shared for the GPs involved, and four meetings were hosted with the GPs to facilitate peer group support.	
Guidelines on the use of the new in-practice service were disseminated internally in the participating practices.	

### Ethics and governance

As this was an evaluation of a change in a NHS service, ethics approval was not required. The PCT and Clinical Commissioning Group (CCG) granted approval for the pilot. A hospital based Consultant Cardiologist and a GPwSI provided clinical support. Guidance regarding patient consent was sought from the Research and Development Department at the PCT. All the participants gave their consent, and their anonymity and confidentiality was ensured.

### Design

Throughout a three month pilot, the participating practices changed the care pathway for patients presenting with non-urgent cardiac conditions by providing a new in-practice cardiology service which accepted in-practice referrals. Patients were assessed investigated and managed by the ECR GP instead of being referred directly to the hospital based specialised cardiology service with onward referral made when judged necessary.

A mixed methods design was adopted to assess the impact of the new model, the perspectives and experiences of patients and GPs. In addition GP and hospital activity data were collected and there was a quality review of a case series.

### GP and hospital activity

#### GP activity and hospital activity post pilot

A bespoke Patient Tracker Form was designed to collect information from each participating GP and included: patient contacts; clinic and referral dates; diagnostic tests requested; clinical outcome and additional clinical information. These data were collated and used to inform a post pilot audit to review the extent to which patients seen by the ECR GP during the study were seen subsequently at the hospital. This was achieved by cross checking patient identifiers recorded on the patient tracker with the hospital activity database. Patients were audited for cardiology activity (both inpatient and outpatient) occurring at the local general hospital for 30 months after the pilot.

#### GP referrals

Choose and Book is a national electronic referral service used by the NHS, which gives patients a choice of place, date and time for their first outpatient appointment in a hospital or clinic. This service was used by the ECR GPs to refer patients to hospital. Data extracts for Choose and Book were provided by the PCT for the period of the pilot and compared to the same period of the preceding year.

### Staff experience

All the clinical team, including all ECR GPs, a Consultant Cardiologist and a GPwSI, as well as a GP partner from each participating GP Practice were invited to take part in a semi-structured interview. These were conducted with the written consent of the participants and were audio recorded and transcribed. The project team devised the interview questions to elicit the GPs’ views on the new role. The transcripts were checked for quality by two researchers from the National Institute of Health Research, Collaboration for Leadership in Applied Health Research and Care, for Leicestershire, Northamptonshire and Rutland’s, team. Text was entered onto computer software NVivo to aid data management and analysis of transcripts for themes and sub-themes using thematic analysis [20].

### Patients’ experience

A bespoke questionnaire (See Additional file 1) was developed by the study team to provide a snapshot of the patients’ experience of their consultation with the ECR GP and to assess how it impacted on their understanding of, and ability to manage their heart problem. The patients were also invited to provide further comments on what was good about the new service and how it could be improved. The data were collected during a single month and questionnaires were distributed by the ECR GP to every patient seen for a Cardiology consultation. The questionnaires were analysed using descriptive statistics and content analysis.

### Clinical quality

A clinical panel, consisting of a GPwSI in Cardiology, a Public Health Consultant and a Consultant Cardiologist reviewed a sample of cases from participating GP practices to assess:

a) The appropriateness of the referral against the criteria set

b) The quality of the record keeping by the ECR GP

c) The quality of the assessment by the ECR GP

d) The quality of the management by the ECR GP

e) The risk of harm under the care of the ECR GP

Each reviewer, independently, gave a score from 1 to 4 to each case using the agreed Criteria for Quality Assessment of Medical Records, as a scoring guide (Table 2). The findings of the 35 cases were summarised and a panel meeting was convened to discuss disagreements and to secure a consensus on the final score.

**Table 2:** Criteria for quality assessment of medical records

**Description of criteria assessment**	**Score**	**Number (%) out of 35 cases**
**Assessing the appropriateness of the referral against the criteria set**		
The problem is well within the competency of a typical GP and should not have been referred to the service	1	0 (0%)
The problem should be within the competency of a typical GP but confidence is variable and some referrals are to be expected	2	1 (3%)
The problem should be within the competency of a GP but access to cardiology investigations is required	3	6 (17%)
The problem is beyond the competency of a typical GP and falls within the remit of the service	4	28 (80%)
**Assessing the quality of the record keeping by the ECR GP**		
There is no record of the consultation with the patient and no discharge record	1	1 (3%)
There is an incomplete record of the consultation with the patient	2	4 (11%)
There is a record including history, examination, investigations but the recommendations and/or management plan are inadequate	3	11 (31%)
There is a full record including history, examination, investigations and a management plan	4	19 (54%)
**Assessing the quality of the assessment by the ECR GP**		
There is no record of the consultation with the patient and no discharge record	1	1 (3%)
The assessment of the patient is inconsistent with best practice	2	3 (9%)
The assessment of the patient is consistent with what might be expected of an ECR GP	3	20 (57%)
The assessment of the patient is entirely consistent with best practice for this type of case	4	11 (31%)
**Assessing the quality of the management by the ECR GP**		
There is no record of the consultation with the patient and no discharge record	1	0 (0%)
The management of the patient is clearly inconsistent with best practice	2	1 (3%)
The management of the patient is consistent with what might be expected of an ECR GP	3	25 (71%)
The management of the patient is entirely consistent with best practice for this type of case	4	9 (26%)
**Assessing the risk of harm under the care of the ECR GP**		
There is no record of the consultation with the patient and no discharge record	1	0 (0%)
There are acts of omission or commission that could put patient safety at risk	2	1 (3%)
There are some acts of omission or commission but safeguards are in place to mitigate any risk to the patient	3	6 (17%)
There are no acts of omission or commission and safeguards are in place to mitigate any risk to the patient	4	28 (80%)

The outcome and findings were disseminated to the ECR GPs towards the end of the pilot both to support a reappraisal of the educational needs of the ECR GPs and to inform the specification, delivery and monitoring of such a service going forward.

## Results

Initially, 13 GP practices of 82 in the county (16%) were recruited to participate in the pilot but one practice withdrew and two practices merged, leaving 10 GP practices.

### GP and hospital activity

#### GP activity

The analysed data from the Patient Tracker form revealed that during the clinical period of the pilot the ECR GPs assessed 364 patients in 12 the weeks of the pilot, and the outcome of this consultation was: 49% (177) of all patients recorded were discharged to their own GP; 21% (75) of all patients recorded had a follow up appointment in clinic; while 4% (14) were discharged, because they were not judged to be cardiology patients. 33 (9%) were referred to an outpatient hospital based cardiology service to be assessed and reviewed by cardiac specialists. There remained a small number of patients referred to hospital as emergency admissions and some recorded as unclassified.

Although the data on final diagnosis was not systematically collected, data on the type of diagnostics ordered provided a picture of clinical mix of the patients seen. It revealed that 274 tests were conducted including 172 ECHOs, 13 24 hour blood pressure monitoring, 77 24 hour ECG monitoring, and 12 exercise tolerance tests. This is consistent with the majority of patients being seen for diagnosis and/or management of heart failure and/or atrial fibrillation, but also included patients with syncope, chest pain and hypertension, a position confirmed by a manual review of the forms from which the tracker data was recorded.

#### Post-pilot audit

An audit was conducted post pilot, on all the 364 cases seen in the pilot. The numbers of cases seen as outpatients or inpatients during the next 6, 12 and 18 months remained low with no suggestion of a post study “bulge” in hospital cardiology activity (Table 3).

**Table 3:** Audit results

**Cardiological activity (both outpatient and hospital admission)**	**Number (%) of out the 364 cases**
No record available on databases	9 (2.5%)
Referred by ECR GP to cardiology	40 (11%)
Referred to cardiology outwith ECR	
GP	
in < 6 months	13 (3.6%)
in 6-12 months	14 (3.9%)
in >12 months	12 (3.3%)

#### GP referrals

**Figure 1:**
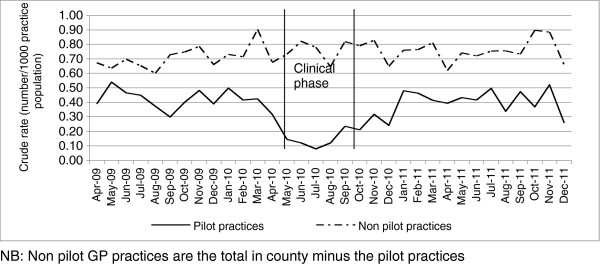
The crude rate trend of choose and book referrals for April 2009 to December 2011.

Comparing the number of Choose and Book cardiology referrals made during the clinical phase of the pilot period, to the same time period in the preceding year revealed that the county witnessed a 12% decrease in referrals. However, the pilot practices observed a 66% decrease in the same period, and then over a three-month period after the pilot had finished the number of referrals returned to pre-pilot levels (Figure 1).

### Staff experience

All of the invited 22 healthcare professionals were interviewed and these were 10 ECR GPs, 10 Partner GPs, and the Cardiologist and the Cardiology GPwSI, therefore representing all of the clinical team’s views. The main themes are presented in Table 4 and the participating GPs’ views on the future of the model and other aspects from the interviews are presented in Table 5.

**Table 4:** The key themes from the interviews with ECR GPs and Partner GPs

**Theme**	**Majority/consensus**	**Minority**
Extended role	The scheme was broadly accepted by the both the partner GPs and ECR GPs. However the need to balance the roles of both a generalist and a specialist were expressed.	ECR GPs expressed feelings of nervousness and anxiety over their newly adopted role
	*“The difficulty is that, we are generalists and asking individual doctors to do more and more in individual clinics would take us away from our role as generalists. I think you have got to be very careful on that front, I think getting that balance right is important” (ECR GP 5)*	
Workload	GPs felt that the extended role had increased their workload but felt it was definitely beneficial to the practice. The increased work was being responsible for more patients, arrangements of tests and follow-up appointments. Some ECR GPs mentioned that the latter was undertaken in their own time. To avoid overbooking the dedicated sessions many GPs accommodated follow-up work amongst their routine work.	Some had support from administrative staff within their practice, which helped to ease their workload
Clinical support	The provision and access to clinical support from the cardiologists was considered to be essential to the new role and for the safety of patients. The model facilitated closer working between primary care and the acute hospital through receiving feedback on clinical triage and clinical queries.	
Benefits	*Patients:* More convenient for patients to be seen at their GP practice; allows patients to receive continuity of care, more accessible for patients. Main benefit was that being the first point of contact for patients, primary care has the advantage of seeing patients early on in their care pathway, allowing them to intervene and manage appropriately at early stages of patients’ illnesses.	Some ECR GPs expressed a desired to pursue a career in cardiology
	*“Found that quite a few patients said it was nice to get the investigations done quickly through [them] and able to come back and talk to [them]” (ECR GP 3).*	
	*GP Practice:* The ECR GP viewed as the practice’s lead in cardiology and colleagues accessed them for advice and guidance on cardiac conditions, which consequently contributed to a decrease in referrals to secondary care.	
	*Personal level:* A positive impact on the GPs’ clinical skills and knowledge which gave them confidence.	

**Table 5:** Participating GPs’ views on the future of the model and other aspects from the interviews

**Theme**	**Majority/consensus**	**Minority**
*Future*		
Sustainability	Several ECR GPs expressed a desire to see the extended GP role continue because of the perceived benefits for patients, clinicians and secondary care. However to be sustainable it would need further resources, including money, skills and time to make it work efficiently. Motivation by the ECR GPs was also central to the future of the new service.	Others felt that the results of an evaluation were needed first to inform the decision to continue the pilot, and that cost-effectiveness also needs to be demonstrated.
		It was felt that patients should be consulted on the implementation of the expansion of the service.
Concerns	The new GP role could change the GPs’ role, from one of a generalist to a specialist in primary care. Furthermore, the transition of care into the community, in the long term, may present a risk of “…*destabilising the hospital sector*” *(ECR GP 8)* due to funds being transferred from the hospital to the community sector.	
Application	The extended GP role model was considered to be appropriate and in line with the new NHS reforms, especially as chronic disease is one of the main problems that the NHS faces in the future.	
*Other aspects*		
Training	All the ECR GPs overwhelmingly appreciated the training course.	
	*“All the additional knowledge and skills just makes me feel a lot more confident that when someone comes along with symptoms of heart failure that I can get straight onto with getting to the cause of the problem and sorting them out properly” (ECR GP 7)*	
Tests accessibility	All ECR GPs stated that having direct access to tests was central to the new service as the immediate access and rapid turnaround of results to the practice was felt to be a key element in the efficiency of the service. The availability of tests enabled the ECR GPs to have more confidence in their decision making in diagnosis and treatment.	
	*“You can’t do it (managing heart failure) without an echo, you can’t diagnose it properly, and so you need the echos” (ECR GP 7)*	

### Patients’ experience

Participating practices agreed to conduct a patient experience survey for a month.

Within that month 54 patients had a consultation with an ECR GP and all were asked to complete the questionnaire. 36 questionnaires were returned, by an almost equal number of males and females (males = 14, females = 15 and 7 = not completed), corresponding to a 66% (36/54) response rate. There was a wide range in the respondents’ age, ranging from 20-29 years group to 90-99 years and the most people were in the 70-79 years group (17%).

The questionnaire was designed to gain the patients’ experience of their consultation with the GP, and is presented below in three categories:

#### Explanation of information

80% (29/36) of the patients felt that the GPs had adequately informed them about the study and 78% (28/36) reported that the tests and the results were clearly explained.

#### During the consultation

72% (26/36) of the patients felt that the GP had put them at they ease during their examination; also that they had been asked about their symptoms and feelings, and that the GP had listened to their concerns and answered their questions. The same percentage of patients was very satisfied with the GPs’ explanation of their heart problem and/or treatment required. 67% (24/36) of the patients felt that their views had been taken into account in relation to making decisions on their care. 75% (27/36) of the patients agreed that the length of the consultation was adequate.

#### Post consultation

After the consultation 72% (26/36) of the patients stated that they understood their condition better and 61% (22/36) felt better able to cope with it. 47% (17/36) of them reported that the consultation had influenced the patients to keep themselves healthy.

From free text responses, patients were pleased with the service and spontaneously drew attention to the benefits of being seen at the surgery, by a familiar doctor without the wait they might anticipate for a hospital appointment and with opportunity for rapid access to tests with results communicated soon after test were concluded.

“Preliminary tests with the GP fine and very prompt - saw him Monday and by Wednesday had blood tests and ECG at the surgery” (Patient survey 9).

Overall the patients were very pleased and satisfied with the service:

“This is an excellent service and should be more readily available” (Patient survey 15).

### Clinical quality

The outcome of the assessment by the clinical review panel against each of the Criteria for Quality Assessment (Table 2) was as follows:

a) *The appropriateness of the referral against the criteria set*

97% (34/35) of cases reviewed were within the remit of the pilot scheme and were considered to meet the ECR GP guidance for the new in-practice cardiology service. In a subset of these cases the panel felt the problem might have been dealt with by a competent GP but access to cardiology tests were required.

b) *The quality of the record keeping by the ECR GP*

In 86% (30/35) cases there was a record including history, examination and investigations, and in 54% (19/35) the record also included a management plan.

c) *The quality of the assessment by the ECR GP*

In 89% (31/35) cases the quality of the assessment by the ECR GPs met the panels’ expectations, but under a third of cases were assessed as consistent with best practice In these cases it was possible to confirm appropriate examination and investigation by scanning different sections of the patient record, but there was no written summary of the findings.

d) *The quality of the management by the ECR GP*

There was appropriate management of patients in the majority of cases. In 97% (34/35) the management was classified as “satisfactory”, but in only 26% (9/35) was this considered to be “best practice”. For most cases the management of the patient was adduced from different sections of the patient record, including the prescribing record and in a minority the ECR GP had concluded with an explicit written management plan for the patient.

e) *The risk of harm under the care of the ECR GP*

On the safety assessment the panel agreed there were no acts of omission or commission in 80% (28/35) of cases. There were a small number of patients where acts of omission or commission were identified, but safeguards, such as ongoing clinical support (Figure 1) were in place to mitigate risk of harm. There was one case where the panel felt that patient safety might have been put at risk and the case was subsequently followed up by the clinical team.

Overall the three clinical reviewers agreed that the standard of clinical practice was acceptable and the scope close to the remit agreed, but that the level of record keeping placed constraints on notes review as an assurance process and needed to be addressed.

## Discussion

This study explored the implications of the innovative Extended Cardiology Role on the GP’s role, patient experience, service delivery, and quality.

### GP’s role

The ECR GP role was broadly welcomed by GPs and the Partner GPs endorsed the new role, as it was considered a welcomed additional resource and an opportunity for professional development. However, caution was expressed about how the ECR GP role may impact on general practice. This concern has been echoed in the work investigating the introduction of the GPwSI role [5,21] which triggered a debate around the specialism of the GP generalist role, which led The Royal College of General Practitioners to issue guidelines on implementing the GPwSI role, which states that “all GPs are specialists in the generalist tradition of Primary Care and that GPs should be a GP first, and then a specialist” [4]. However, Ferrer and colleagues proposed that division of care between generalists and specialists should be reconceptualised from a system perspective [22]. They argue that while referral usually focuses on the narrow question of which type of clinician should see the patient, it makes more sense to optimise the outcomes generated by the generalist–specialist system of care and they called for a greater collaboration between the two areas of care [22]. The piloted model acted as a vehicle to facilitate a closer collaboration between the generalists and specialists through a feedback loop on clinical triage and clinical queries.

A main finding was the impact of the extended role initiative on the workload of the practice. The workload associated with the ECR GP was felt to be significant and some ECR GPs reported using their own time in order to complete the work. Many also said that they had underestimated the administrative aspect of the pilot. This may have been a contributing factor in level of recording keeping that was found in the Clinical Quality review. It was highlighted that having dedicated sessions for the cardiology clinics, access to administrative staff and a clear referral guidelines were vital to manage the workload. There is concern that the initiative will distract the practice from its core business of providing primary care services as previous studies investigating the impact of the GPwSI role have found [5,21].

### Patient experience

One of the benefits presented by the extended role was its impact on patients’ experience of the new service. The findings indicated that patients’ experience of ECR GPs was positive and the provision of care closer to home was welcomed. This is in line with the NHS policy [1], which emphasises the importance of including the patient’s perspective in planned service development. The ECR GPs reported that patients appreciated the new service, emphasising convenience, accessibility, and continuity of care and the main learning from the feedback might be for clinicians to provide sufficient time and adopt consultation approaches that promote shared decision making and supported self-management [23,24].

### Service delivery

At initiation, there was a particular remit for the extended role to deliver improved management of heart failure and atrial fibrillation. Early in the pilot it became clear that work on arrhythmias would extend beyond atrial fibrillation to include assessment of palpitations and syncope and that assessment of intercurrent chest pain might be a useful addition to the scope of the service. All ECR GPs stated that having direct access to tests was central to the new service and a key element in the efficiency of the service. This availability of tests enabled the ECR GPs to have more confidence in their decision making in diagnosis and treatment, improving the primary care management of heart problems and leading to better health outcomes [2].

The notable findings are the strong indications that the GPs in the pilot reported a change in referral activity. A comparison of referral activity in practices during the pilot with the same period in the preceding year revealed a reduction of referrals to hospital based specialist cardiology services, with the post pilot number of referrals returning to pre- pilot levels. The post pilot audit found no evidence for catch up of referrals or emergency presentation to hospital of ERC GP assessed patients in the period following the pilot providing reassurance on the ERC GPs’ ability to take on the new role without detriment to the quality of care and outcomes for the patients. In so far as we could detect, ERC GPs did not miss significant cardiological disease that subsequently required attention in secondary care.

The configuration of this new model of care temporarily changed the care pathway for patients with non-urgent cardiac conditions as it provided an enhanced level of cardiology care with general practice for patients who might otherwise have been referred to a hospital based specialised cardiology service. This new reconfiguration was inspired by the findings of the King’s Fund report [16], which highlighted that a referral management strategy built around peer review, and supported by consultant feedback, with clear referral criteria and evidence-based guidelines is likely to be the most cost and clinically effective.

The support from the NHS Consultant Cardiologist and/or GPwSI in Cardiology was considered essential to the extended role as it provided clinical support and patient safety. Another aspect of this model that was considered to be central by the ERC GPs was that the educational sessions contributed positively towards professional development, increasing confidence and knowledge of heart conditions. This finding is consistent with other evaluation studies [17], which highlighted that a brief educational intervention is the first step in enhancing the capacity of the GP role.

The ECR GP does offer a number of benefits, such as an opportunity to reduce referrals and improve patient experience. If it were to be extended to other practices consideration needs to given to the cost effectiveness of the model and it would need to be carefully monitored in order to fully understand the implications this may have on the hospital sector, as some GPs expressed concerns of the risks of destabilising hospitals’ services through transitioning care into the community.

### Service quality

This was a pilot study with high levels of clinical scrutiny but limited resources for the development and management of systems for monitoring the quality and performance of the scheme. Our assessment of service quality highlighted some areas for improvement and recommendations have been made to commissioners should they wish to explore implementation of the ECR GP model into mainstream practice [25]. These aimed to address clarity in defining the scope of the service, assuring value for money, improving the quality of clinical records and strengthening the patient voice.

The ECR GP service was set up initially to improve management of heart failure and atrial fibrillation in primary care settings, but the scope of practice was seen to include assessment of palpitations and syncope and the assessment of intercurrent chest pain. It was possible that some of the cases seen by ECR GPs might reasonably have been managed by GP partners. As such a review of criteria for ECR referral was conducted and recommendations made that a curriculum be developed for ECR GPs with clear links between the agreed scope of the service, the teaching programme and the evidence base for best clinical practice, with consideration given to the introduction of summative assessment of ECR candidates to assure every clinician was competent to an agreed standard.

The bespoke Patient Tracker form used in the pilot did not include clinical information on the reason for the consultation. This was a drawback as it was not possible to monitor systematically whether all patients seen were appropriate against the criteria set. The curriculum will specify clearly the types of patients that the ECR GP should be seeing and the addition of a suitable dropdown menu to the Patient Tracker would enable commissioners to monitor whether ECR GPs are working within scope as well as providing information on use of diagnostics and outcome of consultations. The Patient Tracker could be part of the development of a robust quality and performance monitoring system that tracks activity, assures performance, and maps to the cost and value of the service.

The quality of record keeping by the ECR GPs was not at the level anticipated. There had been an expectation that ECR GPs would produce a discharge summary including history, examination, investigation and management plan for every patient seen, but this practice was not widely adopted and might need to be specified as a contractual obligation. A recommendation was also made for periodic assessment of clinical performance based on case review as a governance requirement. NICE Guidance on “How to change practice” [26] recommends the use of audit and feedback, observing clinical practice in action; the use of tailored reminder systems and computer-aided decision support systems. Consideration might be given to the design of a bespoke information system that could both provide healthcare professionals with specific information to support decision making and provide a platform for systematic recording of clinical and associated data.

Patient and public involvement in the pilot study was limited to a short patient questionnaire survey of a small sample of patients. The patient voice is critical to the development of any service change and careful consideration is needed on how best to bring the patients’ view into the continued development of the service and how best to assure that the patient experience remains a positive one [27].

### Strengths and limitations of the study

One limitation is that it was a pilot project of 10 practices that was undertaken within one English county, thus the findings cannot be generalised to the population of GPs across the country. There was no comparison group for the patient experience survey, so it is difficult to ascertain the true extent of the patients’ experience and how their experience compared with the service provision at the local hospital. The use of historic data and non-randomized control practices limits the confidence we can place on our measures of impact on Choose and Book referrals to cardiology.

This pilot required the support of the GP Practice Administration staff, although this staff group was not part of the evaluation. A suggestion for a future study would be to include other GP Practice staff groups, such as Practice Managers and Administration staff to ascertain the organisational impact of the new role. Including other stakeholders, such as representatives from hospital, hospital managers, doctors, and specialist cardiology nurses would gain a broader a range of views from which to evaluate the ECR GP.

The GPs who participated in this pilot were selected as they expressed an interest in cardiology management and their positive attitude to the extended role initiative might not be representative of GPs in general. The researchers were aware of this potential bias and aimed to foster an environment that allowed participants to express both negative and positive views and experiences towards the extended role.

## Conclusion

The need for community management of heart conditions has become increasingly vital and the role of the General Practitioners even more fundamental. The extended GP role is broadly welcomed and has the potential to be implemented as model of care that improves the quality of care for patients in primary care. It is also perceived as a professional development opportunity for GPs, and can reduce healthcare utilisation and costs. As this model is developmental further evaluation and consultation with other stakeholders is needed before it is implemented.

## Abbreviations

NHS: National Health Service; GP: General Practitioner; GPwSI: General Practitioner (s) with a Special Interest; PCT: Primary Care Trust. These organisations were responsible for the commissioning local healthcare services in England for populations of about 100,000; CCG: Clinical Commissioning Group. These organisations were set up in shadow form and in April 2013 would formally take on many of the commissioning responsibilities of PCTs; NICE: National Institute for Health and Clinical Excellence; ECR: Extended Cardiology Role; NIHR: National Institute of Health Research; CLAHRC – LNR: Collaboration for Leadership in Applied Health Research and Care - Leicestershire, Northamptonshire and Rutland.

## Competing interests

The authors declare that they have no competing interests.

## Authors’ contributions

The first draft of the paper was prepared by LP, and then all authors contributed to its development and completion. All authors read and approved the final manuscript.

## Pre-publication history

The pre-publication history for this paper can be accessed here:

http://www.biomedcentral.com/1472-6963/14/205/prepub

## Supplementary Material

Additional file 1 The bespoke patient experience questionnaire. Click here for file

## References

[B1] Department of Health Equity and excellence: Liberating the NHS 2011 London: The Stationery Office

[B2] Fonseca C Diagnosis of heart failure in primary care Heart Fail Rev 2006 14 95 107 10.1007/s10741-006-9481-0 16937029

[B3] Department of Health The NHS Plan: a plan for investment, a plan for reform 2000 London: The Stationery Office

[B4] Department of Health and Royal College of General Practitioners Implementing a scheme for General Practitioners with Special Interests 2002 London: The Stationery Office

[B5] Gerada C Wright N Keen J The general practitioner with a special interest: new opportunities or the end of the generalist practitioner? Br J Gen Practice 2002 14 796 800 PMC1316080 12392117

[B6] Royal College of General Practitioners and Royal College of Physicians General Practitioners with special interest 2001 London: RCP

[B7] Pinnock H Netuveli G Price D Sheikh A General practitioners with a special interest in respiratory medicine: national survey of UK primary care organisations BMC Health Service Research 2005 14 40 10.1186/1472-6963-5-40 PMC1166553 15921509

[B8] Coast J Noble S Noble A Horrocks S Asim O Peters TJ Salisbury C Economic evaluation of a general practitioner with special interests led dermatology service in primary care BMJ 2005 14 1444 10.1136/bmj.38676.446910.7C 16339217 PMC1315650

[B9] The Department of Health Guidance and competences for the provision of services using practitioners with Special Interests (PwSIs) Cardiology 2003 London: The Stationery Office

[B10] The Department of Health Guidelines for the appointment of General Practitioners with Special Interests in the Delivery of Clinical Services: Cardiology 2007 London: The Stationery Office

[B11] National Institute for Health and Clinical Excellence (NICE) Chronic heart failure: Management of chronic heart failure in adults in primary and secondary care CG108 This replaced the previous July 2003 CG5 Guidelines 2010 [http://www.nice.org.uk/cg108]

[B12] National Institute for Health and Clinical Excellence (NICE) Atrial fibrillation: The management of atrial fibrillation 2006 Manchester: National Institute for Health and Clinical Excellence

[B13] NHS The Information Centre for Health and Social Care Hospital Episode Statistics Online: Hospital Outpatient Activity. Summary report. 2011-12 [https://catalogue.ic.nhs.uk/publications/hospital/outpatients/hosp-outp-acti-11-12/hosp-outp-acti-11-12-summ-repo-rep.pdf]

[B14] Daniel A Moss M Wilshere S Improving Cardiology referrals from primary to secondary care. Project report 2010 South East Wales Cardiac Network [http://www.wales.nhs.uk/sites3/page.cfm?orgid=490&pid=50325]

[B15] The King’s Fund Referral Management: lessons for success 2010 London

[B16] Royal College of General Practitioners GP with a Special Interest (GPwSI) accreditation[http://www.rcgp.org.uk/clinical-and-research/clinical-resources/gp-with-a-special-interest-gpwsi-accreditation.aspx]

[B17] Clarke D Smith G: Peake JA Trauer T McCall L Blashki G Piterman L Evaluation of a short course in psychiatry for general practitioners Australasian Psychiatry 2006 14 76 80 10.1080/j.1440-1665.2006.02250.x 16630204

[B18] NHS Northamptonshire Duty to Report 1st April 2011 – 31st March 2012 [http://www.miltonkeynes-northamptonshire.nhs.uk/modules/downloads/download.php?file_name=2791]

[B19] Scott I What are the most effective strategies for improving quality and safety of health care? Internal Medicine Journal 2009 14 389 400 10.1111/j.1445-5994.2008.01798.x 19580618

[B20] Ritchie J, Lewis J Qualitative Research Practice: A guide for Social Science Students and Researchers 2005 London: Sage

[B21] Moffat MA Sheikh A Price D Peel A Williams S Cleland J Pinnock H Can a GP a generalist and a specialist? Stakeholders views on a respiratory General Practitioner with a special interest service in the UK BMC Health Services Research 2006 14 62 10.1186/1472-6963-6-62 16734893 PMC1524758

[B22] Ferrer RL Hambidge SJ Maly RC The essential role of generalists in health care systems Annals on Internal Medicine 2005 14 691 699 10.7326/0003-4819-142-8-200504190-00037 15838088

[B23] Elwyn G Edwards A Kinnersley P Shared decision making in primary care: the neglected second half of the consultation British Journal of General Practice 1999 14 477 482 10562751 PMC1313449

[B24] Neuner-Jehle S Schmid M Gruninger U The “Health Coaching” programme: a new patient-centred and visually supported approach for health behaviour change in primary care BMC Family Practice 2013 14 1 100 10.1186/1471-2296-14-100 23865509 PMC3750840

[B25] Rogers S Shribman J Sprigings D Evaluating the Enhanced Cardiology Role. Initial pilot study report for commissioners 2012 Northampton: Northamptonshire County Council Public Health

[B26] National Institute for Health and Clinical Excellence (NICE) How to change practice. Understand, identify and overcome barriers to change 2007 Manchester: National Institute for Health and Clinical Excellence

[B27] Craig GM Involving users in developing health services. Representation is not enough; voices must be translated into action BMJ 2008 14 286 287 10.1136/bmj.39462.598750.80 18230645 PMC2234534

