# Supplementary material for: A study of role expansion: a new GP role in cardiology care
Source: BMC Health Serv Res. 2014 May 6;14:205. doi: 10.1186/1472-6963-14-205 (PMC4048052; doi:10.1186/1472-6963-14-205)
Supplement: Additional file 1 — The bespoke patient experience questionnaire. [file 1472-6963-14-205-S1.doc]

**Additional file 1. The bespoke patient experience questionnaire.**

**Enhanced GP Role in Cardiology Study**

**Patient Experience Questionnaire**

Dear Patient,

We would be very grateful if you would complete this survey about your recent experience during the Enhanced GP Role in Cardiology study.

The doctors involved in the study want to provide the highest standard of care. Feedback from this survey will enable them to identify areas that may need improvement should the service be implemented elsewhere in the county. Your opinions are therefore very valuable and will be added to those of other patients, so your replies will not be identifiable.

Please answer ALL questions that apply to you. There are no right or wrong answers.

We have provided a paid envelope for the return of your questionnaire.

Thank you.

**1. Your regular GP (the GP that you usually see)**

**1.1** **Did your regular GP explain that you were being invited to take part in a study**?

Yes, the explanation was clear □

Yes, but the explanation could have been clearer □

No explanation was given □

**1.2** **Were you given written information regarding the study by your regular GP?**

Yes, the explanation was clear □

Yes, but the explanation could have been clearer □

No explanation was given □

**2. Your Tests**

**2.1** **Were you given information about the cardiology tests by the Enhanced Role GP?**

Yes, the explanation was clear □

Yes, but the explanation could have been clearer □

No explanation was given □

**2.2** **Were the test results explained to you by the Enhanced Role GP?**

Yes, the explanation was clear □

Yes, but the explanation could have been clearer □

No explanation was given □

**3. The Enhanced role in Cardiology GP**

| **Thinking about your consultation with the Enhanced Role GP, how do you rate the following?** | **Poor** | **Fair** | **Good** | **Very good** | **N/A** |
| --- | --- | --- | --- | --- | --- |
| **3.1** How thoroughly the doctor asked about your symptoms and how you are feeling? | □ | □ | □ | □ | □ |
| **3.2** How well the doctor listened to what you had to say? | □ | □ | □ | □ | □ |
| **3.3** How well the doctor put you at ease during your physical examination? | □ | □ | □ | □ | □ |
| **3.4** How much the doctor involved you in decisions about your care? | □ | □ | □ | □ | □ |
| **3.5** How well the doctor explained your heart problem or any treatment you may need? | □ | □ | □ | □ | □ |
| **3.6** The amount of time the doctor spent with you during the consultation(s)? | □ | □ | □ | □ | □ |
| **3.7** The doctor’s patience with your questions or worries? | □ | □ | □ | □ | □ |
| **3.8** The doctor’s caring and concern about you? | □ | □ | □ | □ | □ |

**4. Your Feelings**

**We are interested in any other comments you may have. Please feel free to write them here…**

Is there anything particularly good about the Enhanced GP Role in Cardiology study?

Is there anything that could be improved?

Any other comments?

| **After seeing the Enhanced Role GP in the cardiology clinic do you feel…** | **Much more than before the visit** | **A little more than before the visit** | **The same as before the visit** | **Less than before the visit** |
| --- | --- | --- | --- | --- |
| **4.1** able to understand your problem(s) or illness? | □ | □ | □ | □ |
| **4.2** able to cope with your problem(s) or illness? | □ | □ | □ | □ |
| **4.3** able to keep yourself healthy? | □ | □ | □ | □ |
|  |  |  |  |  |

**5. About you**

**In order for us to understand what type of patients were seen during the pilot and why the pilot was or was not seen as successful the following information would be useful:**

**5.1** Are you… Male □ Female □

**5.2** How old are you? _________________years old

**5.3** Which ethnic group do you belong to?

White □ Mixed □

Black or Black British □ Chinese □

Asian or Asian British □ Other ethnic group □

**Thank you for completing this questionnaire.**

Please use the pre-paid envelope to return the questionnaire to:

Name and address deleted intentionally
